# Neighborhood environment perceptions associate with depression levels and cardiovascular risk among middle-aged and older adults: Data from the Washington, DC cardiovascular health and needs assessment

**DOI:** 10.1080/13607863.2020.1793898

**Published:** 2020-07-21

**Authors:** Marcus R. Andrews, Joniqua Ceasar, Kosuke Tamura, Steven D. Langerman, Valerie M. Mitchell, Billy S. Collins, Yvonne Baumer, Cristhian A. Gutierrez Huerta, Amit K. Dey, Martin P. Playford, Nehal N. Mehta, Tiffany M. Powell-Wiley

**Affiliations:** a Social Determinants of Obesity and Cardiovascular Risk Laboratory, Cardiovascular Branch, Division of Intramural Research, National Heart, Lung, and Blood Institute, National Institutes of Health, Bethesda, MD, USA;; b Section of Inflammation and Cardiometabolic Diseases, National Heart, Lung, and Blood Institute (NHLBI), National Institutes of Health, Bethesda, MD, USA;; c Intramural Research Program, National Institute on Minority Health and Health Disparities, National Institutes of Health, Bethesda, MD, USA

**Keywords:** Neighborhood environment, psychosocial health, cardiovascular disease, depression, inflammatory markers

## Abstract

**Objectives:**

Little is understood about associations between neighborhood characteristics and depression, a cardiovascular disease (CVD) risk factor, in diverse populations. We examined relationships between perceived/objective neighborhood characteristics, depression, and CVD markers within the Washington, DC CV Health/Needs Assessment, an evaluation among predominantly African-American (AA) adults in resource-limited DC communities.

**Method:**

Factor analysis of overall neighborhood environment perception (NEP) identified three NEP sub-scores:1) violence; 2) physical/social environment; 3) social cohesion (higher score = more favorable perception). Objective neighborhood characteristics were measured by geospatially-derived scores of walkability, transportation, and crime. Depression was defined by the revised Center for Epidemiologic Studies Depression Scale (CESD-R). We used linear-regression modeling to examine neighborhood measures and CESD-R associations. To investigate a subsequent connection with CVD risk, we examined relationships between CESD-R and CVD-associated cytokines in a population subset.

**Results:**

Participants (*N* = 99; mean age = 59.06; 99% AA) had a mean CESD-R score 5.8(SD = 8.88). In adjusted models, CESD-R scores decreased by 0.20 units (*p* = 0.01) for every overall NEP unit-increase. Perceived physical/social environment (β = −0.34, *p* = 0.04) and social cohesion (β = −0.82, *p* = 0.01) were related to CESD-R while perceived violence was not (β = −0.28, *p* = 0.1). Of objective neighborhood environment measures (i.e. walk, transit, bike, personal crime, and property crime scores), only property crime score was associated with depression (β = 4.99, *p* < 0.03). In population subset (*n* = 42), higher CESD-R associated with higher IL-1β (β = 21.25, *p* < 0.01) and IL-18 (β = 0.006, *p* = 0.01).

**Conclusion:**

Favorable neighborhood perceptions are related to lower depressive symptoms in a predominantly AA cohort from Washington, DC resource-limited communities. Neighborhood perceptions appear to be strongly associated with depressive symptoms compared to objective characteristics. Increasing CESD-R scores were related to higher pro-inflammatory markers. Improving neighborhood perceptions may be beneficial to psychological well-being and CV health for urban minority residents.

## Introduction

Depression is projected to be one of the leading causes of disability worldwide by 2030 (Mathers & Loncar, 2005). An estimated 16.2 million adults in the United States (6.7% of U.S. adults) have had at least one major depressive episode within the past year (Health, 2017). In recent years, research has begun to elucidate the biological mechanisms that link depression to cardiovascular disease (CVD), and depression remains a risk factor for CVD independent of hypertension, diabetes mellitus, and cigarette smoking (Dhar & Barton, 2016). In 2016, roughly 5% of African American adults indicated that they had experienced a major depressive episode within the last year, compared to 7.4% of white adults (Health, 2017). Despite a lower prevalence of depression, it has been reported that Blacks are more likely to have higher chronicity of depression (R. K. Bailey, Mokonogho, & Kumar, 2019; Breslau, Kendler, Su, Gaxiola-Aguilar, & Kessler, 2005; Williams et al., 2007). Furthermore, depression often goes undiagnosed, and subsequently untreated, in Blacks compared to non-Hispanic Whites (Williams et al., 2007). People with depression have a 50% higher risk of cardiovascular disease (DE Hert et al., 2011). Data that explores these relationships among Black populations are scant; however, data conducted on African American samples show that the risk of cardiovascular disease as a result of depression to be between 30% and 59% compared to those who do not have depression (Copeland et al., 2017; O’Brien et al., 2015). The terms Black and African American may be used interchangeably in this manuscript to refer to persons of the African Diaspora.

Despite having lower rates of diagnosed mental illness than other racial/ethnic groups, African Americans tend to be exposed to social (e.g.discrimination), economic (e.g.perceived barriers to professional advancement), and built environment (e.g.crime, noise) factors that may act as risk factors for psychiatric disorders (Firebaugh & Acciai, 2016; Health, 2017; Hudson, Neighbors, Geronimus, & Jackson, 2012; Simning, Van Wijngaarden, & Conwell, 2012; Ward & Mengesha, 2013). Furthermore, research has identified socioeconomic disparities in depression prevalence, which may be related to environmental exposures (Blair, Ross, Gariepy, & Schmitz, 2014; Hudson et al., 2012). Insufficient data regarding the prevalence of depression in African American communities and optimal treatment approaches for African Americans likely contribute to underdiagnosis and inadequate treatment of depression in African Americans (Sohail, Bailey, & Richie, 2014).

Existing research suggests a link between residential environments and mental health (Kim, 2008; Olsen, Dundas, & Ellaway, 2017). Cutrona et al. asserted the importance of investigating the role of neighborhood environmental context in depression by highlighting three main points (Cutrona, Wallace, & Wesner, 2006). First, people are unaware that their surroundings are impacting their health and subsequently blame themselves. Second, those from other communities are unaware about how structurally disadvantaged neighborhoods impact people’s health. Lastly, given these relationships, there may be more of a need to deal with some of the community-level stressors to which an individual is exposed than to treat each impacted individual separately (Cutrona et al., 2006). Current literature has focused on specific indicators of neighborhood in relation to depression, including crime, social cohesion/disorder, and the physical environment (Choi, Kim, DiNitto, & Marti, 2015; Echeverría, Diez-Roux, Shea, Borrell, & Jackson, 2008; Johns et al., 2012; Miles, Coutts, & Mohamadi, 2012; Perez et al., 2015; Simning et al., 2012; Wilson-Genderson & Pruchno, 2013a). Specifically, higher levels of neighborhood violence and less favorable perceptions of neighborhood safety were associated with an increase in depressive symptoms among adults 50–74 residing in New Jersey (Wilson-Genderson & Pruchno, 2013a). Increased social cohesion, as defined by residents in a community knowing each other well and being willing to help each other, has been associated with decreases in depressive symptoms in some studies, but others have shown no relationship (Choi et al., 2015; Echeverría et al., 2008; Perez et al., 2015). Among a national sample of African Americans and Caribbean Blacks, less favorable perceptions of neighborhood social disorder may be a risk factor for developing depression for both low and middle income respondents (Hastings & Snowden, 2019a). However, few studies have examined different types of neighborhood perceptions or both perceived and objective neighborhood characteristics in relation to depression within a single cohort(Mair et al., 2015).

There is also an imperative to examine the relationship between neighborhood environment and depression in the context of CVD risk. Prior studies support a link between depression and inflammatory markers, including IL-1β, IL-2, and IL-6, contributing to depression as an independent CVD risk factor (Alesci et al., 2005; Danesh, Collins, & Peto, 1997; Dawood et al., 2007; Leo et al., 2006; Maes et al., 1995; Miller, Stetler, Carney, Freedland, & Banks, 2002; Saran, Puri, & Agarwal, 2012). Additionally, depressive symptoms are more likely to be associated with an increased risk of coronary heart disease or revascularization among African Americans compared to whites (Sims et al., 2015). However, prior studies examining the relationship between neighborhood characteristics and depressive symptoms have typically not linked these symptoms to CVD risk.

### Conceptual framework

**Figure 1.**
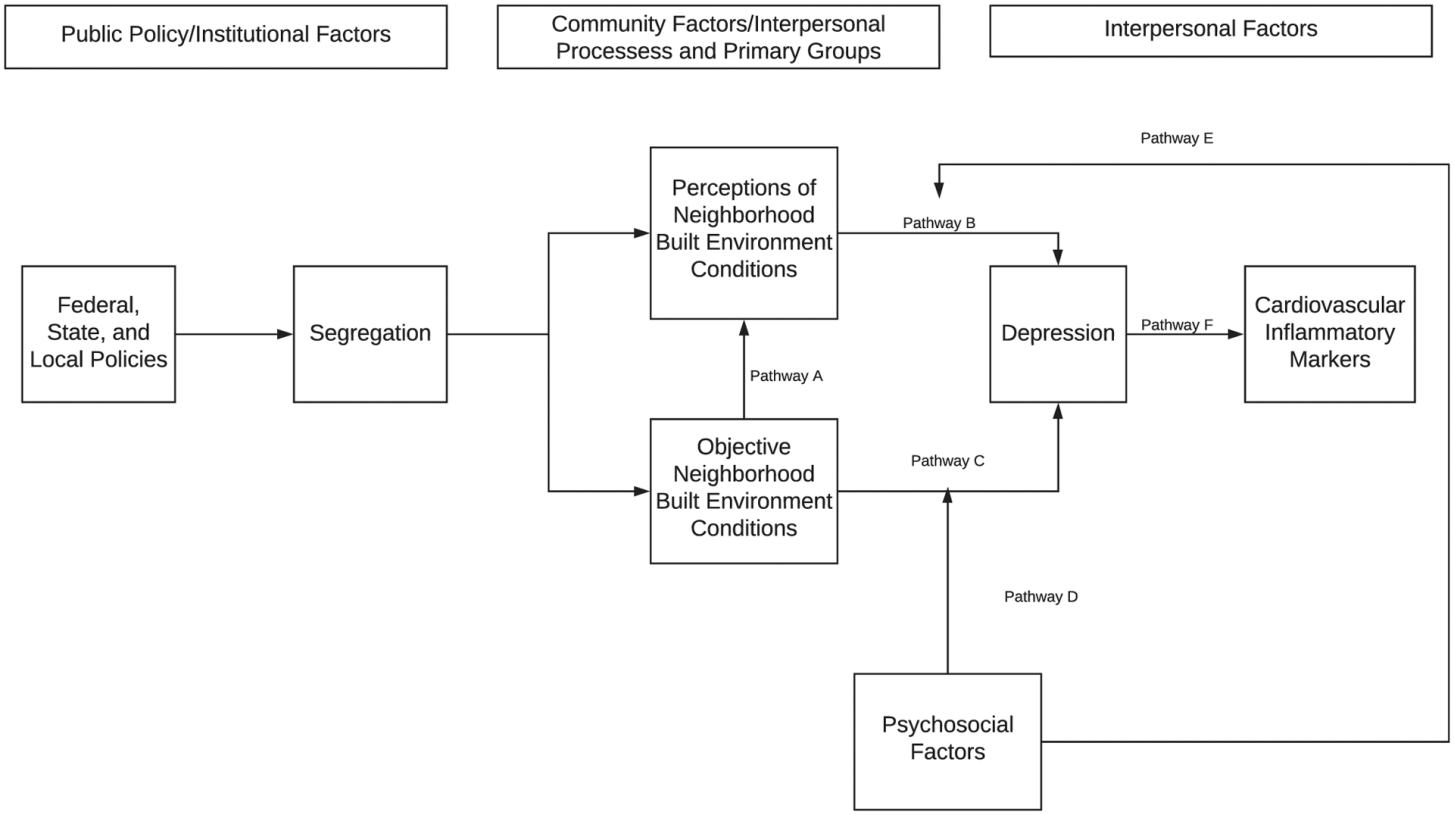
Conceptual Model examining the Proposed Linkages between Neighborhood Environments, Depression, and Cardiovascular Markers.

This manuscript seeks to examine the relationships between neighborhood context, depression, and cardiovascular inflammatory markers using the Socioecological Model proposed by Kenneth McLeroy and colleagues (Mcleroy, Bibeau, Steckler, & Glanz, 1988) (Figure 1). This model states that there are five distinct levels that impact individual outcomes including intrapersonal factors, interpersonal processes and primary groups, institutional factors, community factors, and public policy (Mcleroy et al., 1988). The proposed conceptual model was amended from three existing models (Chang-Martinez, Ahmed, & Ruby, 2017; Lytle, 2009; Schulz et al., 2005). Overarching federal, state, and local policies influenced the racial and ethnic composition of neighborhoods leading to segregated neighborhoods (Rothstein, 2015). Segregated neighborhoods in turn results in disparities in both the social and built environmental conditions which may impact an individual’s wellbeing (Osypuk, Diez Roux, Hadley, & Kandula, 2009; Suglia et al., 2016). Segregated neighborhoods ultimately impacts the objective neighborhood characteristics through producing inequalities in the built (i.e.neighborhood aesthetics, dietary retailers, recreational facilities) and social (crime/safety) environmental conditions (Osypuk et al., 2009; Suglia et al., 2016). Objective neighborhood characteristics, specifically neighborhood walkability, has been cited as potential factor that influences depression among a variety of populations including African Americans (Berke, Gottlieb, Moudon, & Larson, 2007; James, Hart, Banay, Laden, & Signorello, 2017; Orstad et al., 2018). Walkability and urban conditions have also been linked to deleterious mental health conditions through increased population density/overcrowding, social disorganization, street connectivity, and increases in air pollution (Basner et al., 2014; Cho et al., 2014; Dean & Murray, 2005; Galea, Uddin, & Koenen, 2011; James et al., 2017; Marshall, Brauer, & Frank, 2009). Furthermore, objective measures of neighborhood crime have been linked to depression and cognition among older adults (Joshi et al., 2017; Latkin & Curry, 2003; Wilson-Genderson & Pruchno, 2013b). Given that there may be discordance between how people define their neighborhood and publicly available administrative-unit data, incorporating neighborhood perceptions data may capture information about the built environment that more traditional objective measures may not capture (Plunkett, Abarca-Mortensen, Behnke, & Sands, 2007; Roosa, White, Zeiders, & Tein, 2009). Furthermore, research has found that crime indirectly influences depression through individual perceptions around safety and violence (Curry, Latkin, & Davey-Rothwell, 2008a; Wilbur et al., 2009). More recent research has linked neighborhood perceptions of neighborhood social disorder with depressive symptoms among African Americans and Caribbean Blacks (Hastings & Snowden, 2019b). Additionally, psychosocial factors may be potential mediators that alter the link between neighborhood characteristics and depressive symptoms.

### Aims

Therefore, this manuscript has four specific aims based on the proposed conceptual framework: a) examine the relationship between neighborhood environment perceptions and depression, b) examine the relationship between objective measures of neighborhood environment and depression, c) examine the attenuating effects of psychosocial factors on the modeled relationship between neighborhood environment perceptions and depression as an exploratory aim to examine psychosocial factors as potential confounders, and d) examine the relationship between depression and cardiovascular disease among an urban, African American cohort.

## Materials and methods

### Study population

The Washington, DC Cardiovascular Health and Needs Assessment (DC-CHNA) recruited participants from faith-based institutions within DC Wards 5, 7, and 8 to evaluate CV health factors, psychosocial factors, cultural norms, and neighborhood environment characteristics in a predominantly African-American population from at-risk Washington, DC area communities. This assessment served as a preliminary step in the development of a community-based behavioral change intervention to improve CV health in faith-based communities in underprivileged wards of DC (Thomas et al., 2017; L R Yingling et al., 2016). The DC-CHNA data collection events occurred in partnering community-based churches, during which participants went through six stations to complete the assessment, including a station for completing the study survey instrument, as previously described (Yingling et al., 2017). The survey instrument was self-administered with research team members available for assistance to participants who had clarifying questions. The DC-CHNA was approved by the National Heart, Lung, and Blood Institute (NHLBI) Institutional Review Board (NCT01927783). Informed consent was obtained from each study participant. Additional details about the design and recruitment process have been previously reported (Yingling et al., 2016; Yingling et al., 2017). Of the 100 enrolled DC-CHNA participants, *n* = 99 provided initial study data through self-administered surveys. Participants in the DC-CHNA were then recruited to the NIH Clinical Center for additional cardiometabolic phenotyping. All DC-CHNA participants were offered a chance to come to the NIH Clinical Center; 58 participants declined or never responded to the offer, and 42 participants were enrolled into a separate clinical protocol for cardiometabolic testing for those at risk for CVD (ClinicalTrials.gov NCT01143454) which was approved by the National Heart, Lung, and Blood Institute (NHLBI) Institutional Review Board. Informed consent for both the DC-CHNA and the cardiometabolic testing protocols was obtained for all the participants in these studies.

### Study definitions and measurements

#### Neighborhood environment perceptions

As previously described, eighteen questions from the Project on Human Development in Chicago Neighborhoods were used to assess individual perceptions of neighborhood environment focusing on neighborhood violence, sense of community, and physical environmental characteristics (e.g.perceived presence of trash/litter, excessive noise, traffic congestion, sidewalks, and recreation areas; perceived overall violence) (Ahuja et al., 2018). Principal components factor analysis with varimax (orthogonal) rotation was used to create the factor sums as previously described (Ahuja et al., 2018; Estabrooks, Lee, & Gyurcsik, 2003). The scores were standardized on a 5-point scale, where higher scores indicated a more favorable perception of a neighborhood. Principal components analysis with varimax rotation was performed yielding three factors: 1) Neighborhood Violence (e.g.witnessing or hearing about fights with weapons, robberies, gang related activities), 2) Neighborhood Physical/Social Environment (e.g.presence of trash/litter, excessive noise, traffic congestion, sidewalks and recreation areas; perceived overall violence), and 3) Neighborhood Social Cohesion (e.g.feelings of closeness to one’s neighbors). A total perception score and factor scores were calculated for each participant. For each factor, a higher score indicated a more favorable perception of one’s neighborhood (Ahuja et al., 2018).

#### Objective measures of neighborhood environment

Walk Score is a publicly available tool that is used to measure different aspects of walkability including retail access, population density, block length, and intersection density around a particular address (Score, 2019). Several studies have established the validity of the Walk Score metric to assess the conditions around a provided address (Barnes, Winters, Ste-Marie, McKay, & Ashe, 2016; Duncan, Aldstadt, Whalen, & Melly, 2013; Duncan, Aldstadt, Whalen, Melly, & Gortmaker, 2011; Koohsari et al., 2018) Transit Score measures how well a particular address has access to public transportation looking specifically at frequency of public transportation, distance to the nearest transit stop, and the type of route (bus, train). Bike Score assigns a score to an area based on the presence of bike lanes, hills, connectivity, and the frequency of bikers. For Walk Score, Transit Score, and Bike Score, increases in scores are associated with more favorable environments. Personal Crime and Property Crime score data are collected from police departments where they are then sorted by severity and distance. The population per capita rate is calculated and then compared to inter-city scores and then assigned a letter grade (Score, 2019). The grades for both Personal Crime and Property Crime scores were coded so that increases in personal crime and property crime indicate a less favorable score.

#### Outcomes

Depressive symptom scores were assessed using the Revised Centers for Epidemiologic Studies Depression Scale (CESD-R) (Maruish, 2004). The 20-item scale measures depressive symptoms within nine key areas as defined by the American Psychiatric Association Diagnostic and Statistical manual, which include sadness, loss of interest, appetite, sleep, thinking/concentration, guilt, tiredness, movement, and suicidal ideation. A total score of 16 or higher may be suggestive of clinical depression.

#### Inflammatory markers from cardiometabolic testing

For the subset of the original population that underwent subsequent cardiometabolic testing (*n* = 42), our health care provider team performed a physical examination on each of these patients and obtained each patient’s demographic data, clinical history, and anthropometric measurements. Blood samples were collected after an overnight fast and analyzed for basic chemistry, complete lipid profile, and high sensitivity C-reactive protein at the NIH Clinical Center. Interleukins (IL)-1β, IL-6, IL-18 were measured using a multiplex ELISA (Meso Scale Diagnostics, Rockville MD, USA), as described previously (Bastarache, Koyama, Wickersham, & Ware, 2014).

#### Covariates

Demographic information on sex and household income were self-reported. Height was measured using a stadiometer (Perspective Enterprises, Portage, MI) and weight was measured using a calibrated scale (Doran Scales, Inc., Batavia, IL), and body mass index (BMI) was calculated from the height and weight obtained during the DC-CHNA using the following formula “weight (kg) / height(m)2”(Centers for Disease Control & Prevention, 2017). Marital status was self-reported as either “married, divorced/separate/widowed, or single”. The Atherosclerotic Cardiovascular Disease (ASCVD) risk score was calculated for each participant based on age, race, sex, smoking status, serum total cholesterol, serum HDL cholesterol, blood pressure, diabetes status, and treatment for these conditions (Wilson et al., 1998).

Chronic stress, social isolation, social life/social support, and the spiritual health locus of control were considered as psychosocial factors that may serve as confounders within our regression models. The Chronic Stress Scale consists of 51 items about life situations where respondents are asked to respond with “not true (0), somewhat true (1), or very true (2)” (Hamilton et al., 2011; Turner, Wheaton, & Lloyd, 1995). Social life/social support and social isolation were measured as subscales of the Chronic Stress Scale, a validated metric that is designed to be broken into 13 sub-scales (Hamilton et al., 2011; Turner, Wheaton, & Lloyd, 1995; Wheaton, 1994, 1997). Subscales for the Chronic Stress Scale have been used in prior studies (Scott, Whitehead, Bergeman, & Pitzer, 2013). Social life/social support was measured using questions from sub-scales of the Chronic Stress Scale (Hamilton et al., 2011). Participants were asked to rate their levels of agreement with the following four statements: “1)You have to go to social events alone and you don’t want to, 2)Your friends are a bad influence, 3) You don’t have enough friends, and 4)You don’t have time for your favorite leisure time activities” by selecting one of the following: not true (0), somewhat true (1), or very true (2) (Hamilton et al., 2011). Social isolation was also measured using a sub-scale of the Chronic Stress Scale. Participants were asked to rate their levels of agreement with the statement “You are alone too much.” by selecting one of the following options not true (0), somewhat true (1), or very true (2). We used the 13-item validated Spiritual Health Locus of Control (SHLOC) scale: the active SHLOC subscale comprises 11 items with a possible overall score between 11–55 and the passive SHLOC sub-scale comprises 2 items with a possible overall score between 2–10. Active SHLOC is the belief that both the individual and God are in control of their health, whereas Passive SHLOC is the belief that God alone controls their health (Holt, Clark, Kreuter, & Rubio, 2003).

The Neighborhood Deprivation Index (NDI) was created using variables from the 2010 United States Census Bureau, as previously described (Andrews et al., 2020; Diez Roux, Borrell, Haan, Jackson, & Schultz, 2004; Lian, Struthers, & Liu, 2016; Powell-Wiley et al., 2020). Ultimately, 11 of 13 variables around employment/occupation, education, housing, and wealth/income were used to create the NDI scores on a census tract level (% Employed in Management, % Unemployment, % High School Graduates, % Bachelor’s Degree or Higher, % Households Without Telephones, % Households Without Plumbing, Household Income, Home Value, % Family Poverty, % Public Assistance, % Female-Headed Household, % Owner Occupied Housing Units, % Housing Units Receiving Interest/Dividends, Rental Income). Each variable was standardized and reverse coded, as necessary. Oblique rotation was applied (minimum loading score of 0.40; minimum eigenvalue of 1) and a Cronbach’s alpha was applied with a minimum consistency for each factor of 0.70. The sum of the standardized variables was used to create the NDI values. This method has been shown to be associated with CVD risk and has been recommended for use by expert consensus (Saelens et al., 2018). Higher NDI scores symbolize a more socioeconomically deprived neighborhood.

### Statistical methods

Descriptive data for the study population are presented as means with standard deviations for continuous variables and percentages for categorical variables. Linear regression modeling was used to determine the relationships between the overall neighborhood perception scores, each of the perception factor scores, and the depressive symptoms score. Linear regression was also used to determine relationships between the objective neighborhood measures (Walk Score, Transit Score, Bike Score, Personal Crime score, Property Crime score) and CESD-R score. Models were adjusted for household income, Neighborhood Deprivation Index (NDI), BMI, and ASCVD risk score. Since age sex, and race are considered in the ASCVD calculation, these demographic characteristics are not added as separate covariates in the model. As an exploratory analysis, the additional psychosocial variables (chronic stress, social life/social support, social isolation, and SHLOC) were included as separate covariates in the model to assess for attenuation of the fully adjusted model by these additional psychosocial factors. As an additional exploratory analysis, within the sub-sample of the population who underwent detailed cardiometabolic testing, (*n* = 42), linear regression models were used to estimate the relationships between depressive symptoms scores and levels of inflammatory cytokines. These models were adjusted for ASCVD risk score, household income, NDI, and BMI. P-values ≤0.05 were considered significant. SPSS Statistics 24 (IBM Corp., Armonk, N.Y.) was used in data analyses.

## Results

The study sample within the DC Cardiovascular Health and Needs Assessment (*n* = 99) was 78.8% women, with a mean age of 59.06 (SD 12.14) years. About 59% of the population had a yearly household income under $60,000 and 40% were married. The mean depressive symptoms scores were 5.8 (8.88 SD) and the mean total neighborhood perception score was 56.13 (12.59 SD). Factor 1 (Neighborhood Violence), Factor 2 (Physical and Social Environment), and Factor 3 (Social Cohesion) had mean scores of 19.16 (6.17 SD), 23.40 (6.60 SD), and 12.55 (4.06 SD) respectively (Table 1).

**Figure 2.**
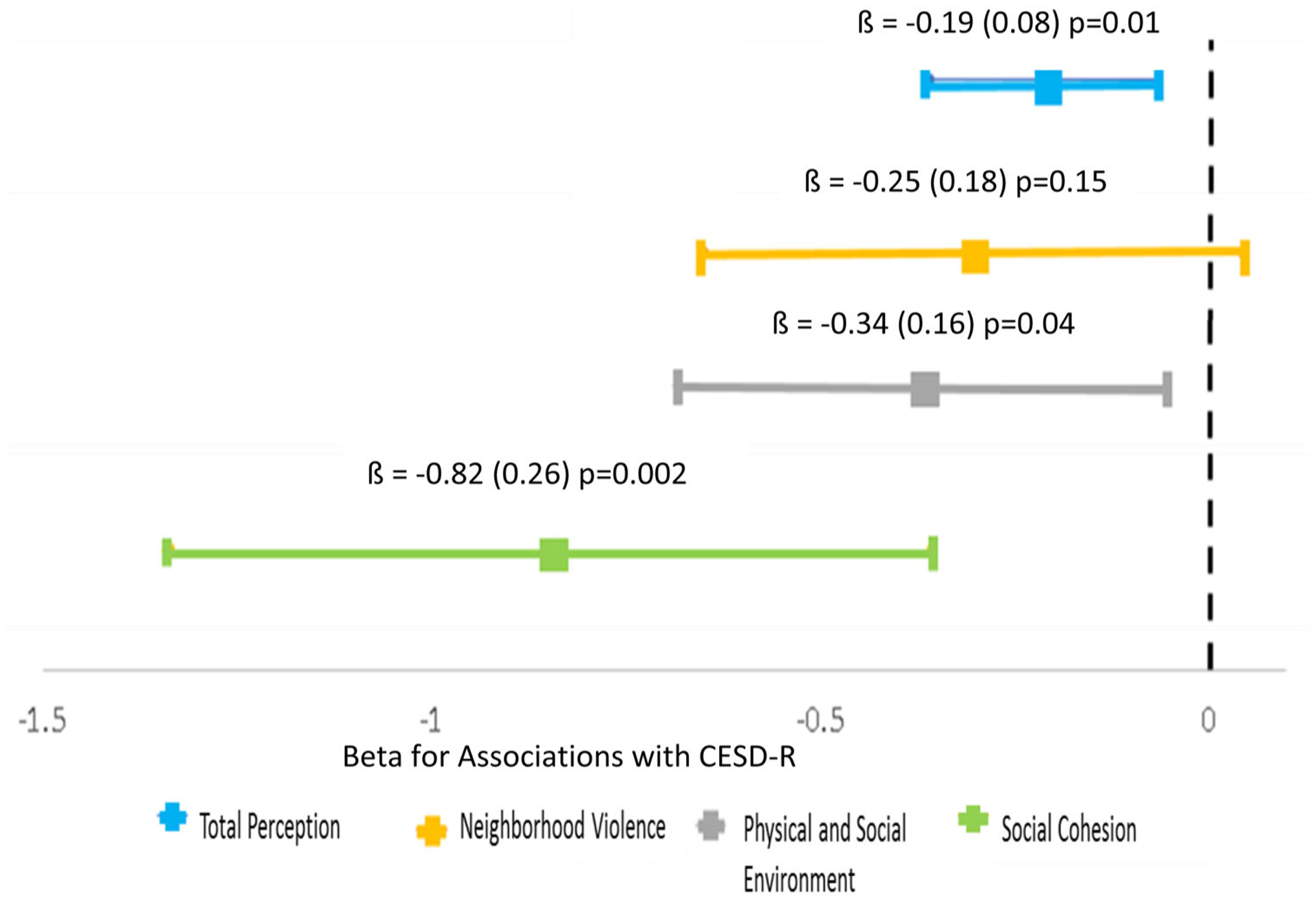
More Favorable neighborhood perception factors associate with Lower CESD-R scores among participants from the Washington, DC Community Health and Needs Assessment (*N* = 99). *Note: Models adjusted for: Household Income, Neighborhood Deprivation Index, Body Mass Index, and ASCVD risk score (95% Confidence Interval).

We found an inverse relationship between favorable neighborhood perceptions and depressive symptom scores. In models adjusting for household income, NDI, BMI, and ASCVD risk score, better overall neighborhood perceptions were related to a 0.20 unit decrease in depressive symptom scores (Total Perception Score: β= −0.19 *p* = 0.01). More favorable perceptions of neighborhood physical/social environment and social cohesion were associated with decreasing depressive symptom scores; however, neighborhood violence was not associated with depressive symptom scores (neighborhood violence: β=−0.25, *p* = 0.15; physical/social environment: β=−0.34, *p* = 0.04; social cohesion: β=−0.82, *p* = 0.002) (Figure 2). These patterns of associations remained consistent when adjusting for additional psychosocial factors including chronic stress, social isolation, and active and passive SHLOC. However, the association between neighborhood perception scores related to physical/social environment and depressive symptom scores was no longer significant when adjusting for social life/social support (Table 2).

When looking at objective measures of the environment, more favorable scores around Walk Score (β = 0.05, *p* = 0.20), Transit Score (β = 0.03, *p* = 0.66), Bike Score (β = 0.05, *p* = 0.30), and Personal Crime score (β = 2.72, *p* = 0.30) were not significantly related to depressive symptom scores. However, less favorable scores for Property Crime (β = 4.94, *p* = 0.02) was related to increases in depressive symptoms, even after adjusting for covariates (Table 3). This pattern remained when adjusting for chronic stress, social isolation, and spiritual locus of control as psychosocial factors. However, the relationship between property crime score and depressive symptom scores was attentuated when adjusting for social life/social support (Table 4).

Among the subset of the study population (*n* = 42) that underwent cardiometabolic testing, there was a statistically significant relationship between depressive symptom scores and inflammatory cytokines. We observed significant increases in IL-1β (20.42, *p* < 0.01) and IL-18 (0.006, *p* < 0.01) for every unit increase in depressive symptom score while non-significant associations were observed for IL-6 (β = 0.43, *p* = 0.55) and CRP (β= −0.033, *p* = 0.71) (Table 5).

## Discussion

In this study, we examined the relationship between perceived and objective neighborhood environment characteristics and depressive symptom scores using the Washington, D.C. Cardiovascular Health and Needs Assessment; we also examined the subsequent association between depressive symptom scores and pro-inflammatory markers associated with CVD in this population. As we hypothesized, more favorable overall neighborhood perception was related to lower depressive symptom scores, particularly for perceptions about physical and social environment and social cohesion. Additionally, these relationships were unlikely to be attenuated when examined in tandem with other psychosocial factors. However, we did not see a consistent relationship between objective measures of environment and depressive symptom scores. Less favorable property crime scores were associated with increases in depressive symptom scores. Our findings also indicate a relationship between increases in depressive symptom scores and increases in CVD-related inflammatory markers. Our research contributes to the growing knowledge regarding the relationship between neighborhood environment, mental health, and CV health among urban, lower income African Americans.

Individuals’ perceptions of their surrounding environment may not always be concordant with objective measures of the same environment; hence, as our study suggests, it is important to examine the ways in which both individuals’ perceptions and more objective measures of environment associate with health behaviors and outcomes (E. J. Bailey et al., 2014). Cutrona, Wallace, and Warner discussed the importance of considering neighborhood context and characteristics in relation to depression as less favorable neighborhood characteristics may interfere with individuals’ ability to form bonds with one another, thus exacerbating the risk for depressive symptoms (Cutrona et al., 2006). Our findings support their hypothesis as we have illustrated an inverse relationship between individual perceptions of one’s environment and depressive symptom scores, with the strongest relationship being found for perceived social cohesion. Consequently, strengthening the social networks within these DC-area communities could be especially important in reducing depressive symptom scores and improving CV health. When examining the relationship between neighborhood perceptions, psychosocial factors, and depressive symptoms, we also found that adding social life/social support to the models attenuated the relationship between perceived physical/social environment and depression scores. These findings suggest that fostering social life/social support may be important in countering the relationship between unfavorable neighborhood perceptions of the built environment and depressive symptoms.

Research supports a relationship between neighborhood context and depression through exposure to socioeconomic and sociodemographic factors and a lack of protective social relationships; however, our study contributes to the literature because limited data exists around this topic for urban, resource-limited African Americans (Blair et al., 2014; Matheson et al., 2006). Our results found a positive relationship between less favorable objective measures of property crime and depression, but did not find a significant relationship between less favorable measures of personal crime and depression. In a previous study, researchers utilized path analysis to explore the relationship between neighborhood violence and depressive symptoms in a sample of current and former drug users in Baltimore, Maryland (Curry et al., 2008b). Their findings did not find a direct pathway between neighborhood violent crime and depressive symptoms. However, their findings suggested that neighborhood violent crime impacted depressive symptoms indirectly through perceptions of neighborhood disorder and experiences with neighborhood violence (Curry et al., 2008b). In some ways, our findings support this prior study because we saw that perceptions about social cohesion and physical/social environment were more strongly associated with depressive symptoms than perceptions of neighborhood violence or objective personal crime. Thus, fostering social support and social networks may help limit the impact of both perceived and objective neighborhood characteristics on depression.

Collectively, our findings also support a need to consider multi-level interventions that address both individual-level treatment approaches and neighborhood environment conditions in reducing depression. For example, structurally disadvantaged communities are faced with numerous stressors including unstable housing conditions, unemployment or underemployment, and environmental hazards, which all may impair one’s cognitive function but also impact perceptions about neighborhood environment (Cutrona et al., 2006). Alleviating community-level stressors due to adverse built environment characteristics may improve cognitive function of community members and ultimately lower risk for depression (Dubowitz et al., 2015). While changes to the built environment represent a potential avenue for intervention, they must be undertaken with caution as they risk contributing to gentrification, which can adversely affect populations with imposed vulnerabilities and actually exacerbate depressive symptoms through changes in the cultural composition of their neighborhoods and subsequent changes in perceived social cohesion (Mair et al., 2015), (Curry et al., 2008b). Future work should further explore the role of gentrification in mental health and CV risk, particularly for vulnerable populations.

Our study suggests that only changing the physical environment through interventions is not enough and targeting perceptions of neighborhood environment may be most beneficial in reducing depressive symptoms. Researchers in Germany examined whether perceptions of neighborhood environment changed after a pedometer intervention (Wallmann, Spittaels, De Bourdeaudhuij, & Froboese, 2012). Ultimately, their findings suggested that increasing the exposure individuals have to a particular neighborhood environment may lead to changes in their perceptions around their neighborhood environment (Wallmann et al., 2012). Additionally, targeting social support may be beneficial in changing the relationship between neighborhood perceptions and measures of depression. Researchers have employed community-based participatory research paradigms to address the lack of neighborhood social cohesion and safety within an under-served Birmingham, Alabama neighborhood. The collaboration yielded a “Get to Know Your Neighbor Day” which served to promote community cohesion through hosting both adults and children for a day of activities and relationship building. Respondents indicated that perceiving that the neighborhood was improving and consistent interactions with their neighbors fostered a higher level of social cohesion (Bateman et al., 2017). These types of interventions can be considered and evaluated in other urban communities, including the target areas of the Washington DC CV Health and Needs Assessment.

Our study also contributes to the scant research on the relationship between depression and inflammatory markers specifically within African American communities. Depression has been linked to inflammation and subsequent CVD through both pathophysiologic dysregulation and worsening health behaviors (Dhar & Barton, 2016; Raison & Miller, 2011). Specifically, people who suffer from depression experience higher levels of psychological stress, which may lead to activation of both the sympathetic nervous system and the hypothalamic-pituitary-adrenal axis (Dhar & Barton, 2016). Under prolonged stress responses, the sympathetic nervous system is activated without the expected activation of the parasympathetic nervous system (Won & Kim, 2016). This can result in central nervous system dysregulation leading to endothelial dysfunction, coronary vasoconstriction, platelet activation, and the release of proinflammatory cytokines, ultimately leading to CV events (Dhar & Barton, 2016).

Within the subset of our population that underwent additional cardiometabolic phenotyping, we found a significant relationship between depression and inflammatory markers. Our findings are consistent with previous work regarding the link between depression and CV health (Dhar & Barton, 2016; Miller et al., 2002). Miller et, al., found that among healthy adults without any acute infectious disease or chronic medical illnesses, those who met clinical depression criteria had significantly higher levels of both CRP and IL-6 (Miller et al., 2002). In a study of African Americans recruited from the Washington, D.C. area, there was a stronger relationship between increased CRP levels and depression for those who experienced greater hostility and had lower educational attainment (Mwendwa et al., 2013). However, among our participants, we found that increases in depressive symptom scores were linked to significant increases in IL-1β and IL-18, but not IL-6 and CRP, even after adjusting for household income, CVD risk markers, and NDI. The lack of association between depression symptoms and IL-6 or CRP in our cohort as compared to other cohorts may reflect differences in the distribution of age, sex, or body composition between the cohorts. Ultimately, we demonstrate that individual perceptions of neighborhood environmental conditions may impact risk for depression for this urban, predominantly African American cohort, which may contribute to increases in pro-inflammatory markers, putting this cohort at risk for future CV events. These results yield insights into mechanisms by which neighborhood factors and environmental exposures “get under the skin” and impact CV health outcomes.

Strengths of this study include use of validated survey measures for multi-dimensional perceptions of neighborhood characteristics, psychosocial factors, and depressive symptoms (Bell et al., 2011). However, limitations of our work must be noted. Due to the cross-sectional nature of this study, causal effects are unable to be explored. Given that our study relies on self-reported data, some of the responses may be subject to social desirability bias. Having more robust measures of crime and violence exposure may illuminate these relationships further. We are unable to do formal mediation analyses in the study looking at psychosocial factors as potential mediators of the relationship between neighborhood characteristics and depression with the limited sample size, and these analyses could be considered in the future work with larger epidemiologic data-sets. Additionally, the psychometric tools used for measuring social life/social support and social isolation must be considered in the context of chronic stress and may be more optimally measured by psychometric scales that measures specific subtypes of social support and social isolation. Furthermore, given our specific population and small sample size, these results may not be generalizable to other African Americans or other populations who reside in urban cities across the United States.

## Conclusions

In conclusion, we examined the relationship between perceived and objective neighborhood characteristics, depressive symptoms, and inflammatory markers in a predominantly African American, urban population from faith-based institutions in resource-limited Washington, DC communities. We found that more favorable perceptions around neighborhood physical and social environment were associated with fewer depressive symptoms. However, perceived neighborhood violence was not significantly related to depression scores. Neighborhood environment perceptions were more strongly associated with depressive symptoms than objective measures of neighborhood characteristics. Additionally, depressive symptoms were associated with pro-inflammatory markers of CV risk. Future research should examine causal pathways by which adverse neighborhood conditions promote depression and subsequent CV risk within lower income minority communities and identify opportunities for multi-level interventions. This exploratory study should be replicated in larger epidemiologic studies to explore the potential mechanisms by which neighborhood built environment and perceptions influence depression and CVD risk (Tamura et al., 2020). Building upon this study, an additional study that examines specific pathways through which neighborhood built environment characteristics are related to CV risk through depression could help develop additional models around neighborhoods and mental health functioning.

## Figures and Tables

**Table 1.** Sociodemographic, clinical, psychosocial, and neighborhood envioronment characteristics of participants from the Washington, DC Community Health and Needs Assessment (*N* = 99).

	All individuals *(N* = 99)
**Demographic Characteristics**	
Female, N (%)	78 (78.79)
Mean Age, Years (SD)	59.06 (12.13)
Race/Ethnicity	
African American	98 (99%)
White	1 (1%)
**Education, N (%)**	
<High School	9 (9.18)
High School	10 (10.2)
Some College	34 (34.69)
College	45 (45.92)
**Yearly Household Income, N(%)**	
<$60,000	40 (58.82)
$60,000–99,999	28 (41.18)
**Marital Status N (%)**	
Married	40 (40%)
Divorced/Separated/Widowed	11 (11%)
Single	14 (14%)
**Clinical Characteristics**	
Systolic Blood Pressure mmHg, Mean (SD)	131.51 (20.07)
Diastolic Blood Pressure mmHg, Mean (SD)	78.94 (14.52)
Total Cholesterol mg/dL, Mean (SD)	194.92 (45.16)
HDL Cholesterol mg/dL, Mean (SD)	53.64 (16.87)
LDL Cholesterol mg/dL, Mean (SD)	110.96 (43.77)
ASCVD Risk Score, Mean (SD)	12.19 (11.47)
Body Mass Index kg/m^2^, Mean (SD)	32.61 (7.04)
**Psychosocial Measures and Neighborhood Environment Characteristics**	
Depressive Symptom Score, Mean (SD)	5.80 (8.88)
Chronic Stress, Mean (SD)	18.00 (4.51)
Social Isolation, Mean (SD)	0.45 (0.66)
Social life/social support, Mean (SD)	2.52 (1.69)
Active Spiritual Health Locus of Control, Mean (SD)	47.44 (10.13)
Passive Spiritual Health Locus of Control, Mean (SD)	3.49 (1.98)
Total Perception Score, Mean (SD)	56.13 (12.59)
Factor 1: Neighborhood Violence, Mean (SD)	19.16 (6.17)
Factor 2: Physical Environment, Mean (SD)	23.40 (6.60)
Factor 3: Social Cohesion, Mean (SD)	12.55 (4.06)
Walk Score, Mean (SD)	46.10 (27.61)
Transit Score, Mean (SD)	57.03 (19.39)
Bike Score, Mean (SD)	46.03 (21.44)
Personal Crime, Mean (SD)	2.15 (0.88)
Property Crime, Mean (SD)	2.53 (0.86)
Neighborhood Deprivation Index-NDI Mean (SD)	−1.56 (2.69)
**Inflammatory Markers, Mean (SD)** *	
Il-1β pg/mL	0.25 (0.20)
IL-6 pg/mL	4.83 (3.7)
IL-18 pg/mL	1148.24 (440.71)
CRP g/L	4.97 (9.23)

* Note: Inflammatory markers are provided for the population subset (*n* = 42) who underwent cardiometabolic testing.

**Table 2.** Regression Results exploring the Relationship between Neighborhood Perceptions, Psychosocial Factors, and Depressive Symptom Scores: Washington, DC Community Health and Needs Assessment (*N* = 99).

Neighborhood Perception Measure	Model 1		Model 2		Model 3		Model 4		Model 5	
	Depression		Depression		Depression		Depression		Depression	
	β (SE)	p-value	β (SE)	p-value	β (SE)	p-value	β (SE)	p-value	β (SE)	p-value
Total Perception^a^	−0.20 (0.07)	0.004	−0.19 (0.07)	0.005	−0.16 (0.07)	0.02	−0.22 (0.08)	0.004	−0.19 (0.08)	0.015
Factor 1: Neighborhood Violence^b^	−0.28 (0.16)	0.08	−0.26 (0.15)	0.099	−0.24 (0.16)	0.13	−0.28 (0.18)	0.113	−0.24 (0.18)	0.18
Factor 2: Physical and Social Environment^c^	−0.40 (0.14)	0.007	−0.38 (0.14)	0.007	−0.27 (0.15)	0.068	−0.38 (0.16)	0.02	−0.34 (0.17)	0.047
Factor 3: Social Cohesion^d^	−0.68 (0.24)	0.005	−0.66 (0.23)	0.006	−0.63 (0.24)	0.01	−1.01 (0.26)	0.00	−0.81 (0.26)	0.002

Model 1: Adjusted for chronic stress, household income, neighborhood deprivation index, body mass index, and ASCVD risk score.

Model 2: Adjusted for social isolation, household income, neighborhood deprivation index, body mass index, and ASCVD risk score.

Model 3: Adjusted for social life/social support, household income, neighborhood deprivation index, body mass index, and ASCVD risk score.

Model 4: Adjusted for active spiritual health locus of control, household income, neighborhood deprivation index, body mass index, and ASCVD risk score.

Model 5: Adjusted for passive spiritual health locus of control, household income, neighborhood deprivation index, body mass index, and ASCVD risk score. For all models, Beta-coefficient represents unit-change in depressive symptom score for one-unit change in overall perceived neighborhood environment and perceived environment factors.

* Each psychosocial factor was tested as an additional covariate in these regressions examining the relationship between neighborhood perception factors and depressive symptom scores.

a Neighborhood Violence: measures perceptions about safety and violent fights.

b Physical and Social Environment: measures perceptions about presence of trash/litter, excessive noise, traffic congestion, sidewalks, and recreation areas; perceived overall violence.

c Social Cohesion: measures perceptions about similar values, trust, and helpfulness of neighbors.

* Higher Scores = better perceptions.

**Table 3.** Regression results exploring the relationship between objective measures of neighborhood environment and depressive symptom scores: Washington, DC Community Health and Needs Assessment (*N* = 99).

	Depression	
	β(SE)	p-value
Walk Score	0.05 (0.04)	0.20
Transit Score	0.03 (0.07)	0.66
Bike Score	0.05 (0.05)	0.30
Personal Crime score	2.72 (2.58)	0.30
Property Crime score	4.94 (2.10)	0.02

* Notes: Models adjusted for: Household Income, Neighborhood Deprivation Index, Body Mass Index, and ASCVD risk score.

**Table 4.** Regression results exploring the relationship between the active neighborhood checklist, psychosocial factors, and depressive symptom scores: Washington, DC Community Health and Needs Assessment (*N* = 99).

Objective Neighborhood Measure	Model 1		Model 2		Model 3		Model 4		Model 5	
	Depression		Depression		Depression		Depression		Depression	
	B (SE)	p-value	B (SE)	p-value	B (SE)	p-value	B (SE)	p-value	B (SE)	p-value
Walk Score	0.04 (0.03)	0.21	0.04 (0.03)	0.29	0.05 (0.03)	0.13	0.05 (0.04)	0.20	0.05 (0.04)	0.19
Transit Score	0.03 (0.06)	0.06	0.03 (0.07)	0.61	0.08 (0.06)	0.23	0.03 (0.07)	0.67	0.03 (0.07)	0.66
Bike Score	0.06 (0.04)	0.17	0.002 (0.04)	0.96	0.05 (0.04)	0.27	0.05 (0.05)	0.26	0.05 (0.05)	0.28
Personal Crime	3.19 (2.02)	0.12	2.74 (2.36)	0.26	0.79 (2.31)	0.73	3.03 (2.57)	0.25	3.05 (2.74)	0.27
Property Crime	3.94 (1.68)	0.025	5.21 (1.89)	0.01	2.93 (1.98)	0.15	4.92 (2.08)	0.02	5.56 (2.22)	0.02

Model 1: Adjusted for chronic stress, household income, neighborhood deprivation index, body mass index, and ASCVD risk score.

Model 2: Adjusted for social isolation, household income, neighborhood deprivation index, body mass index, and ASCVD risk score.

Model 3: Adjusted for Social life/social support, household income, neighborhood deprivation index, body mass index, and ASCVD risk score.

Model 4: Adjusted for active spiritual health locus of control, household income, neighborhood deprivation index, body mass index, and ASCVD risk score.

Model 5: Adjusted for passive spiritual health locus of control, household income, neighborhood deprivation index, body mass index, and ASCVD risk score. For all models, Beta-coefficient represents unit-change in depressive symptom score for one-unit change in objective environment scores.

* Each psychosocial factor was tested as an additional covariate in these regressions examining the relationship between objective neighborhood measure-sand depressive symptom scores.

**Table 5.** Regression results exploring the relationship between depressive symptom score and inflammatory markers: Washington, DC Community Health and Needs Assessment Sub Study (*N* = 42).

Marker	Depression β (SE)	P-value
IL-1β pg/mL	20.42 (4.07)	<0.01
IL-6 pg/mL	0.430 (0.718)	0.55
IL-18 pg/mL	0.006 (0.002)	<0.01
CRP mg/L	−0.033 (0.087)	0.71

* Note: Models adjusted for ASCVD risk score, BMI, Household Income, and NDI.
